# Role of JNK in a *Trp53*-Dependent Mouse Model of Breast Cancer

**DOI:** 10.1371/journal.pone.0012469

**Published:** 2010-08-30

**Authors:** Cristina Cellurale, Claire R. Weston, Judith Reilly, David S. Garlick, D. Joseph Jerry, Hayla K. Sluss, Roger J. Davis

**Affiliations:** 1 Howard Hughes Medical Institute, Program in Molecular Medicine, University of Massachusetts Medical School, Worcester, Massachusetts, United States of America; 2 Department of Cancer Biology, University of Massachusetts Medical School, Worcester, Massachusetts, United States of America; 3 Department of Veterinary & Animal Science, University of Massachusetts, Amherst, Massachusetts, United States of America; 4 Division of Endocrinology, Department of Medicine, University of Massachusetts Medical School, Worcester, Massachusetts, United States of America; University of Texas MD Anderson Cancer Center, United States of America

## Abstract

The cJun NH_2_-terminal kinase (JNK) signal transduction pathway has been implicated in mammary carcinogenesis. To test the role of JNK, we examined the effect of ablation of the *Jnk1* and *Jnk2* genes in a *Trp53*-dependent model of breast cancer using BALB/c mice. We detected no defects in mammary gland development in virgin mice or during lactation and involution in control studies of *Jnk1^−/−^* and *Jnk2^−/−^* mice. In a *Trp53^−/+^* genetic background, mammary carcinomas were detected in 43% of control mice, 70% of *Jnk1^−/−^* mice, and 53% of *Jnk2^−/−^* mice. These data indicate that JNK1 and JNK2 are not essential for mammary carcinoma development in the *Trp53^−/+^* BALB/c model of breast cancer. In contrast, this analysis suggests that JNK may partially contribute to tumor suppression. This conclusion is consistent with the finding that tumor-free survival of JNK-deficient *Trp53^−/+^* mice was significantly reduced compared with control *Trp53^−/+^* mice. We conclude that JNK1 and JNK2 can act as suppressors of mammary tumor development.

## Introduction

The cJun NH_2_-terminal kinase (JNK) group of signaling enzymes are activated by cytokines/growth factors and also by exposure to environmental stress [1]. Targets of the JNK pathway include members of the activator protein 1 (AP1) group of transcription factors (e.g. cJun, JunB, and JunD). JNK is therefore a major regulatory mechanism of AP-1 dependent gene expression [1]. In addition, JNK can regulate many cytoplasmic and nuclear processes [2]. These studies have implicated the JNK signaling pathway in the regulation of cell growth and cell death [1]. Dysregulation of the JNK pathway may therefore contribute to the development of cancer [3].

The role of JNK in cancer has been studied using mouse models that are JNK-deficient. Two genes (*Jnk1* and *Jnk2*) encode isoforms of JNK that are ubiquitously expressed [1]. *Jnk1^−/−^* mice and *Jnk2^−/−^* mice are viable, but compound mutant *Jnk1^−/−^ Jnk2^−/−^* mice exhibit an early embryonic lethal phenotype [1]. Studies using *Jnk1^−/−^* mice and *Jnk2^−/−^* mice indicate that JNK may have isoform-dependent effects on cancer. Thus, Bcr-Abl-induced lymphoma [4] and carcinogen-induced hepatocellular carcinoma [5] are suppressed in *Jnk1^−/−^* mice. Moreover, carcinogen-induced skin cancer is suppressed in *Jnk2^−/−^* mice [6]. Similarly, important roles for JNK2 have been identified in studies of human glioblastoma, prostate cancer, and lung carcinoma cell lines [7]–[10]. Together, these data confirm that both JNK1 and JNK2 can play roles in tumor development.

The purpose of this study was to test the requirement of JNK1 and JNK2 in a mouse model of mammary carcinoma. Somatic mutation of the human p53 gene (*TP53*) is common in sporadic breast cancer [11]. Furthermore, mammary carcinoma is the most common form of cancer in women with heritable mutations in *TP53* (Li-Fraumeni syndrome) [12]. Initial studies using mouse models demonstrated that *Trp53^−/−^* animals develop lymphoma with high frequency and that *Trp53^−/+^* animals display a moderately broader tumor spectrum with slower onset of disease [13], [14]. Subsequent studies using *Trp53^−/+^* mice on a BALB/c strain background demonstrated that, like humans with Li-Fraumeni syndrome, mammary carcinomas were frequently observed, together with some lymphomas and sarcomas [15]. The BALB/c mouse model can therefore be used to examine *Trp53*-dependent formation of mammary carcinoma.

We report that JNK1 and JNK2 are not required for the development of mammary carcinoma in the *Trp53^−/+^* BALB/c mouse model. In contrast, the tumor-free survival of JNK-deficient *Trp53^−/+^* mice was reduced compared with control *Trp53^−/+^* mice. These data suggest that JNK may partially contribute to tumor suppression.

## Materials and Methods

### Mice

We have described *Jnk1^−^* ^/*−*^ mice [16] and *Jnk2^−^* ^/*−*^ mice [17] on a C57BL/6J strain background [18], and mice with *Trp53* gene ablation [13] on a BALB/cMed strain background [19]. The mice used in this study were backcrossed (ten generations) to the BALB/cJ strain (Jackson Laboratories) and were housed in a facility accredited by the American Association for Laboratory Animal Care (AALAC). The Institutional Animal Care and Use Committee (IACUC) of the University of Massachusetts Medical School approved all studies using animals (Docket A-1032).

### Genotype analysis

Genotype analysis was performed by PCR using genomic DNA as the template. The wild-type *Jnk1* (460 bp) and knockout *Jnk1* (390 bp) alleles were identified using the amplimers 5′-CGCCAGTCCAAAATCAAGAATC-3′, 5′-GCCATTCTGGTAGAGGAAGTTTCTC-3′, and 5′-CCAGCTCATTCCTCCACTCATG-3′. The wild-type *Jnk2* (400 bp) and knockout *Jnk2* (270 bp) alleles were identified using the amplimers 5′- GGAGCCCGATAGTATCGAGTTACC-3′, 5′-GTTAGACAATCCCAGAGGTTGTGTG-3′, and 5′-CCAGCTCATTCCTCCACTCATG-3′. The wild-type *Trp53* (470 bp) and knockout *Trp53* (700 bp) alleles were identified using the amplimers 5′-TATACTCAGAGCCGGCCT-3′, 5′-ACAGCGTGGTGGTACCTTAT-3′ and 5′-CTATCAGGACATAGCGTTGG-3′.

### Analysis of tissue morphology

Mammary gland development was examined in virgin female mice (8 to 10 weeks of age), lactating mice (1 week post partum), and mice with mammary gland involution (pups removed at 1 week post-partum). The fourth inguinal mammary gland pair was dissected from each mouse; one gland was analyzed by whole mount and the other was formalin-fixed and paraffin-embedded.

Whole mounts were performed by spreading the gland on a glass slide and incubation (2–4 hrs.) with Carnoy's fixative (60% ethanol, 30% chloroform, 10% glacial acetic acid). The glands were then incubated with a graded series of 70%, 50% and 25% ethanol (15 mins each), followed by 5 minutes in water and stained with carmine alum overnight. The glands were washed in 70%, 90% and 100% ethanol (15 mins each), two changes of xylene (30 mins), and then mounted with Permount (Fisher Scientific).

Analysis of tissue sections was performed using tissue fixed in 10% formalin for 24 h, dehydrated, and embedded in paraffin. Sections (7 µm) were cut and stained using hematoxylin and eosin (Biocare Medical). Immunofluorescence analysis was performed using de-parafinized sections treated with the endogenous Biotin-Blocking kit (Invitrogen), staining (4°C, 12 h) with biotin-conjugated anti-PCNA (Invitrogen), and the incubation (25°C, 1 hr) with AlexaFluor633-conjugated Streptavidin (Invitrogen). The sections on coverslips were washed and mounted on slides using VectaShield medium containing DAPI (Vector Labs.). Images were examined using a Leica TCS SP2 confocal microscope.

## Results

### Effect of JNK-deficiency on mammary gland development

**Figure 1:**
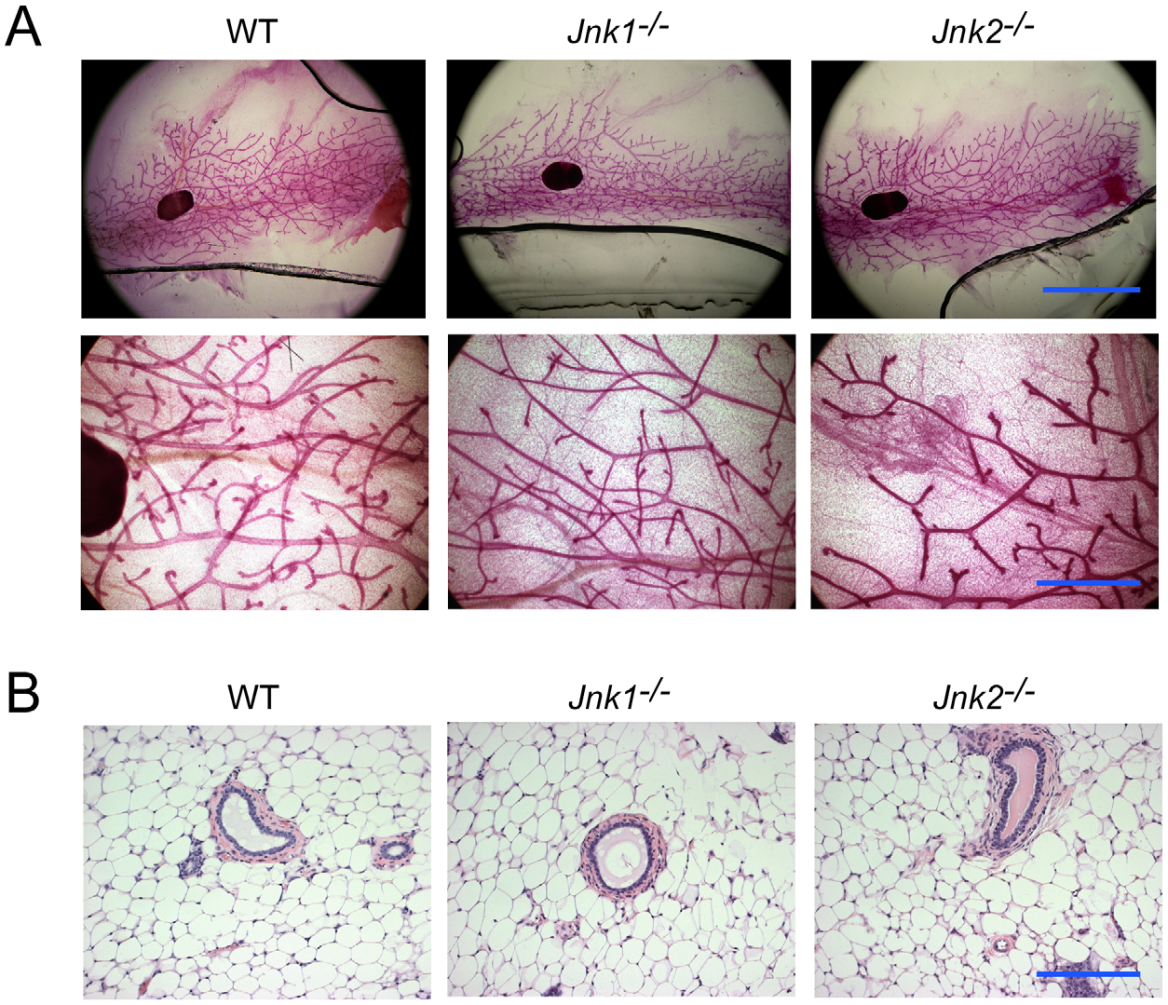
Effect of JNK-deficiency in virgin mice on breast development. **A**) Whole mount preparations of the fourth inguinal mammary gland of 10 week-old female virgin mice were stained with carmine red. Representative images are presented. Scale bar: 5 mm (*upper panel*); 800 µm (*lower panel*). **B**) Sections of the breast tissue were stained with hematoxylin and eosin. Representative images are presented. Scale bar: 100 µm.

We backcrossed *Jnk1^−^* ^/*−*^ mice [16] and *Jnk2^−^* ^/*−*^ mice [17] to the BALB/cJ strain background. To test whether JNK-deficiency altered mammary gland development, we examined *Jnk1^−/−^* and *Jnk2^−/−^* BALB/c mice. No defects were detected in whole mount preparations of fourth inguinal mammary glands of JNK-deficient virgin female mice compared with control mice (Figure 1A). Sections prepared from these mammary glands confirmed that JNK-deficiency did not cause major defects in virgin mammary gland development (Figure 1B).

**Figure 2:**
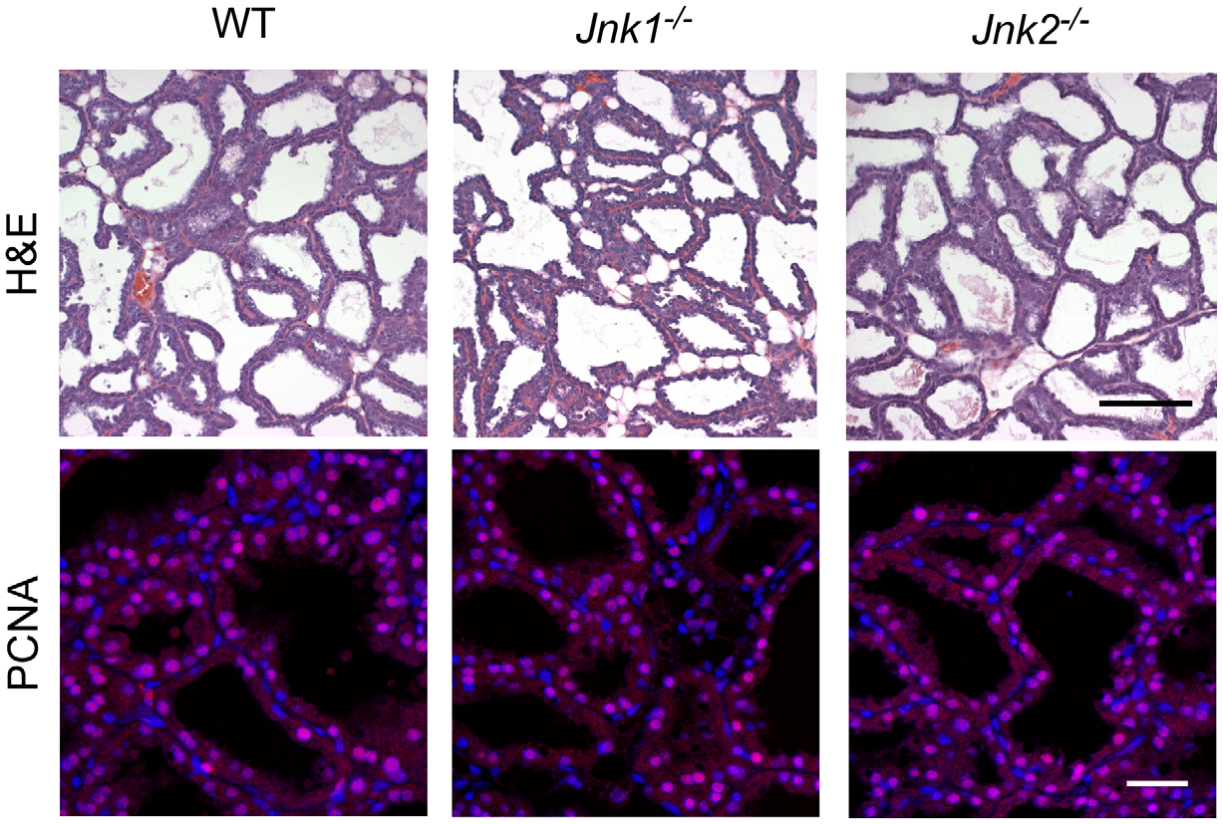
Effect of JNK-deficiency on breast development during lactation. Sections of the fourth inguinal mammary gland of female mice at day 7 post-partum were examined by staining with hematoxylin and eosin (upper panels). Sections were also stained with an antibody to the proliferation marker PCNA (red) and the DNA stain DAPI (blue) (lower panels). Scale bar: 200 µm (*upper panel*); 50 µm (*lower panel*).

Pregnancy causes major changes in mammary gland development, including the formation of alveoli. Sections prepared from the fourth inguinal mammary glands of JNK-deficient lactating mice and control lactating mice were similar (Figure 2). Indeed, sections stained for proliferating cell nuclear antigen (PCNA) indicated that JNK-deficiency did not alter epithelial cell proliferation in the lactating mammary gland (Figure 2).

**Figure 3:**
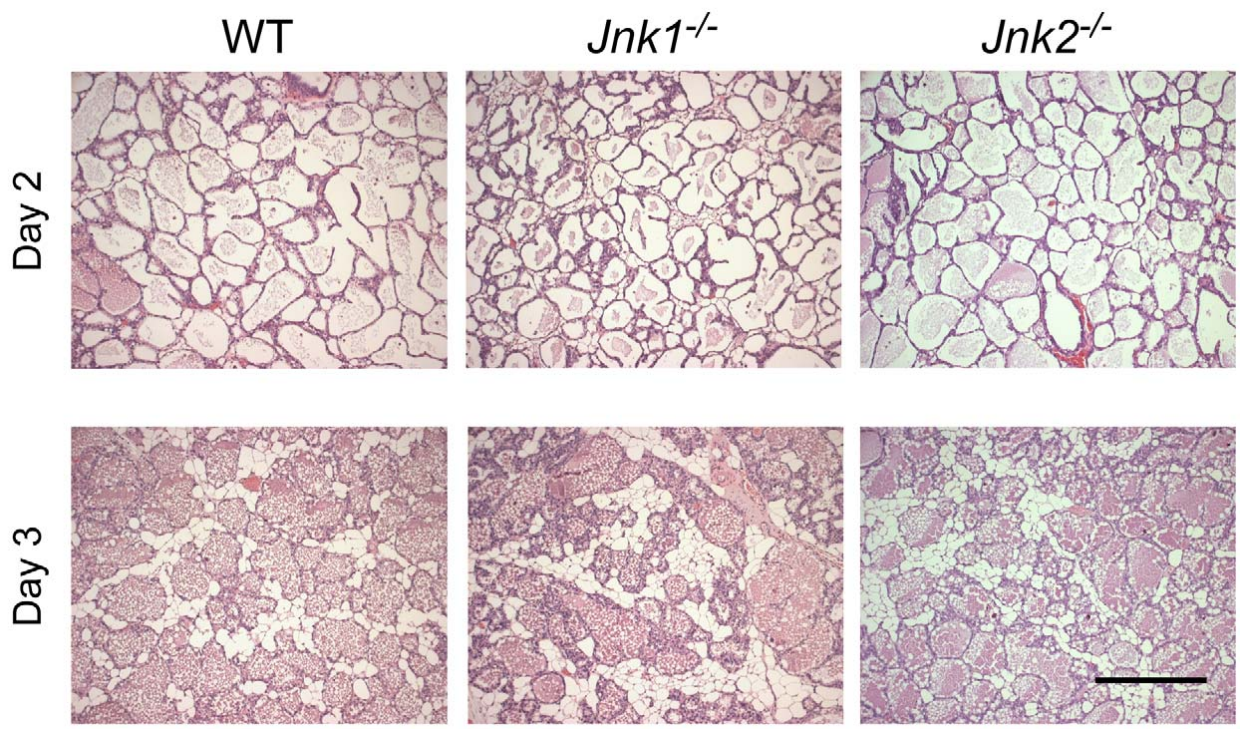
Effect of JNK-deficiency on mammary gland involution. The pups were removed from female mice at day 7 post-partum to induce mammary gland involution. Sections of the fourth inguinal mammary gland were examined at two days or three days post-weaning by staining with hematoxylin and eosin. Scale bar: 200 µm.

Involution of the lactating mammary gland occurs after weaning pups. We compared sections of the fourth inguinal mammary glands prepared on day 2 and day 3 following weaning. No defects in involution were detected in JNK-deficient mice compared with control mice (Figure 3).

Together, these data demonstrate that JNK1-deficiency and JNK2-deficiency did not cause detected changes in mammary gland development. Similarly, no developmental defects caused by JNK1-deficiency or JNK2-deficiency were detected in *Trp53^−/+^* mice.

### Effect of JNK-deficiency on tumor development in Trp53*^−/−^* BALB/c mice

**Figure 4:**
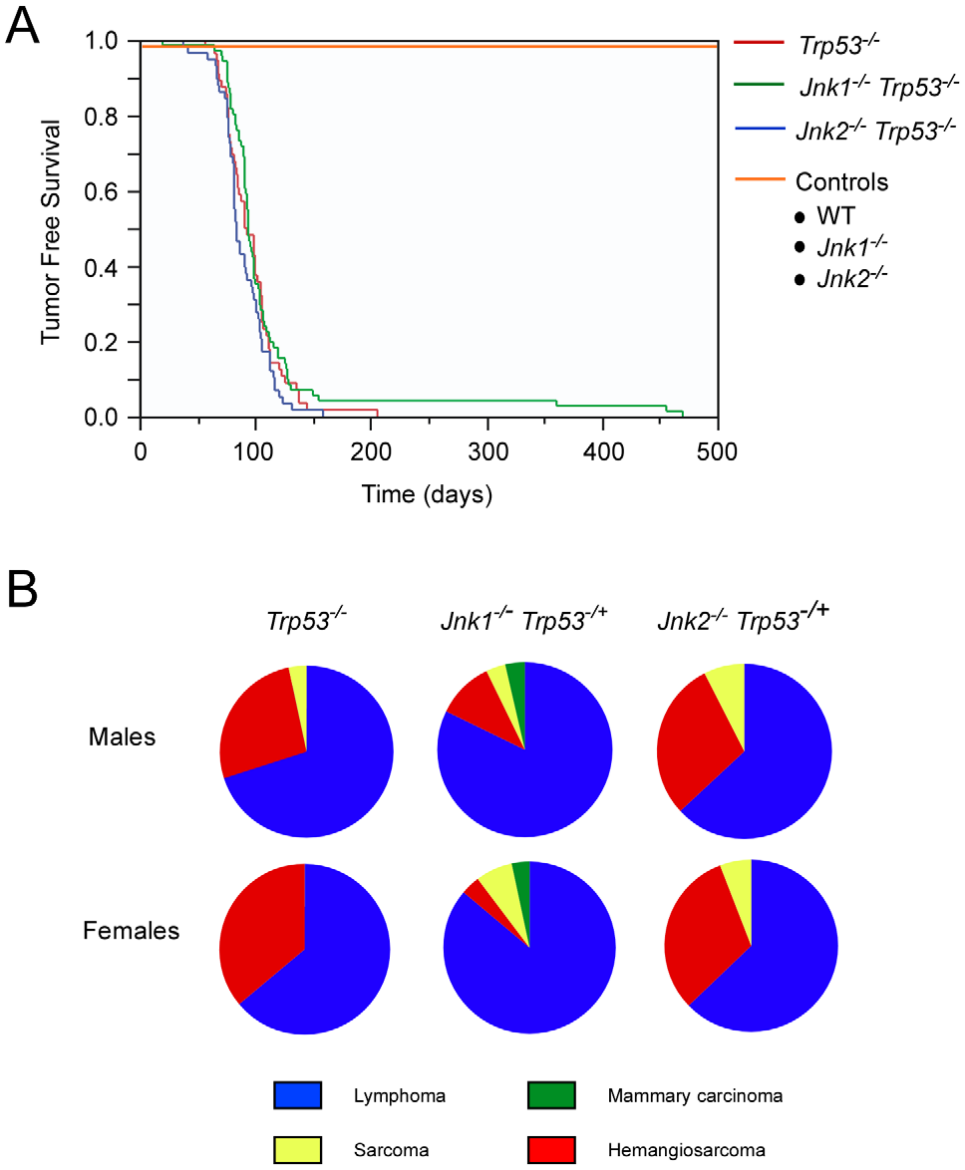
Effect of JNK-deficiency on *Trp53^−/−^* mouse survival. **A**) Kaplan-Meier analysis of the tumor-free survival of wild-type (WT), *Jnk1* ^−/−^, *Jnk2* ^−/−^, *Trp53* ^−/−^, *Jnk1* ^−/−^ *Trp53* ^−/−^, and *Jnk2* ^−/−^ *Trp53* ^−/−^ mice is presented. No statistically significant differences between *Trp53* ^−/−^, *Jnk1* ^−/−^ *Trp53* ^−/−^, and *Jnk2* ^−/−^ *Trp53* ^−/−^ mice were detected (Log-rank test, p>0.05). The data represent groups of 44–62 mice. These groups include equal numbers of male and female mice. **B**) The spectrum of tumors detected in *Trp53* ^−/−^, *Jnk1* ^−/−^ *Trp53* ^−/−^, and *Jnk2* ^−/−^ *Trp53* ^−/−^ mice following euthanasia is presented. No statistically significant differences in the tumor profiles between genotypes were detected using Fisher's exact test.

We examined the tumor-free survival of *Trp53^−/−^* mice, *Jnk1^−/−^ Trp53^−/−^* mice, and *Jnk2^−/−^ Trp53^−/−^* mice on a BALB/c strain background. The mice rapidly developed cancer and died (Figure 4A). No significant differences in tumor-free survival between control and JNK-deficient mice were detected. Pathological examination of the mice demonstrated, as expected, a high incidence of lymphoma (Figure 4B). The second most frequent type of tumor detected in *Trp53^−/−^* mice and *Trp53^−/−^ Jnk2^−/−^* mice was hemangiosarcoma (Figure 4B). In contrast, *Jnk1^−/−^ Trp53^−/−^* mice displayed fewer hemangiosarcomas and a higher incidence of lymphoma compared with *Trp53^−/−^* mice (Figure 4B). These data suggest that JNK1 may influence the tumor spectrum of *Trp53^−/−^* mice. Indeed, a low incidence of mammary carcinoma was observed in both male and female *Jnk1^−/−^ Trp53^−/−^* mice, but not in *Trp53^−/−^* mice or *Jnk2^−/−^ Trp53^−/−^* mice (Figure 4B). The presence of mammary carcinoma in *Jnk1^−/−^ Trp53^−/−^* mice indicates that JNK may be relevant to breast cancer.

### Effect of JNK-deficiency on tumor development in Trp53*^−^* ^/+^ BALB/c mice

**Figure 5:**
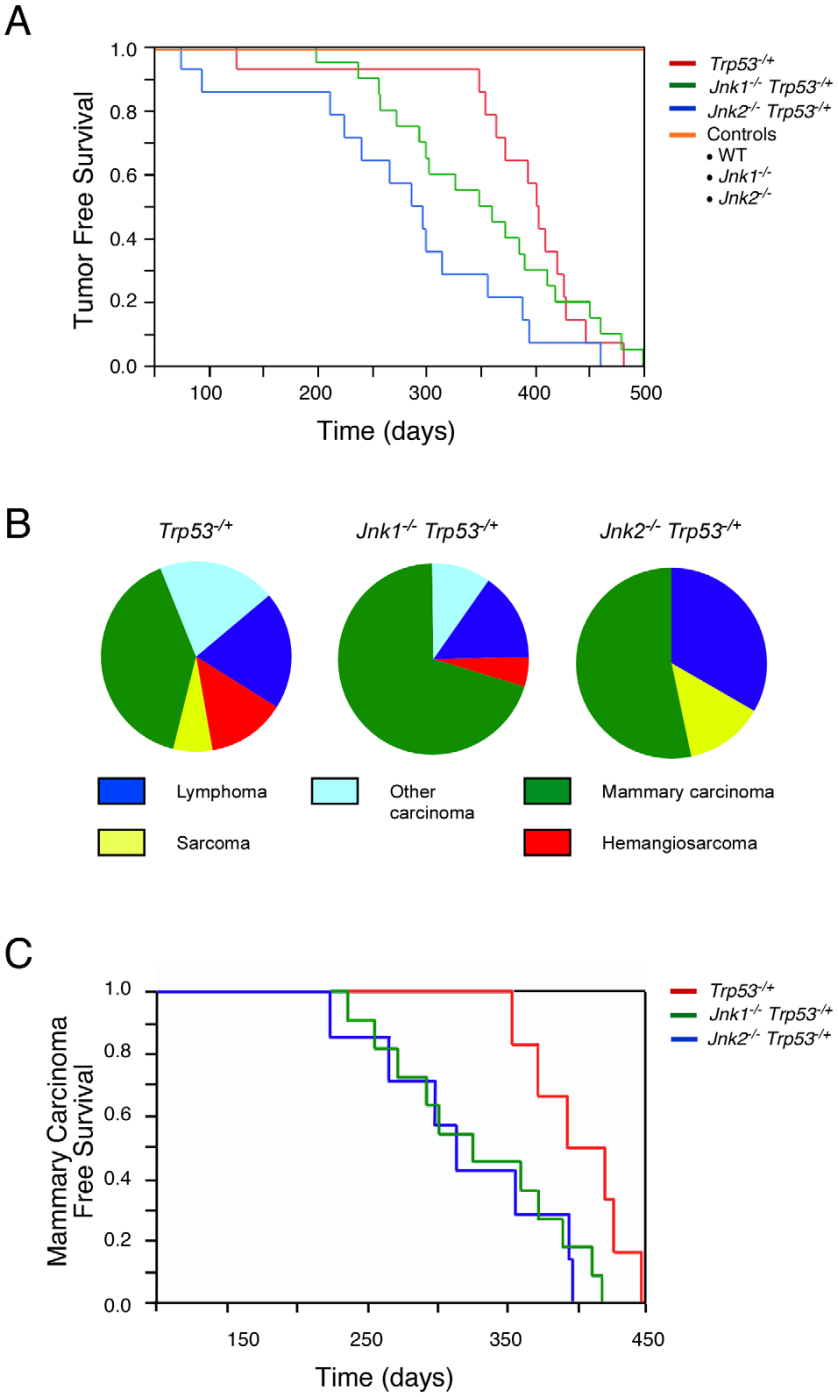
Effect of JNK-deficiency on *Trp53^−/+^* mouse survival. **A**) Kaplan-Meier analysis of the tumor-free survival of wild-type (WT), *Jnk1* ^−/−^, *Jnk2* ^−/−^, *Trp53* ^−/+^, *Jnk1* ^−/−^ *Trp53* ^−/+^, and *Jnk2* ^−/−^ *Trp53* ^−/+^ mice is presented. The survival of *Jnk1* ^−/−^ *Trp53* ^−/+^ mice and *Jnk2* ^−/−^ *Trp53* ^−/+^ mice was reduced compared with *Trp53* ^−/+^ mice (Log-rank test, p = 0.026 and 0.012, respectively). The data represent groups of 14 - 20 female mice. **B**) The spectrum of tumors detected in *Trp53* ^−/+^, *Jnk1* ^−/−^ *Trp53* ^−/+^, and *Jnk2* ^−/−^ *Trp53* ^−/+^ female mice following euthanasia is presented. No statistically significant differences in the tumor profiles between genotypes were detected using Fisher's exact test. **C**) Kaplan-Meier analysis of the mammary carcinoma-free survival of *Trp53* ^−/+^, *Jnk1* ^−/−^ *Trp53* ^−/+^, and *Jnk2* ^−/−^ *Trp53* ^−/+^ mice is presented. Cohorts of *Trp53* ^−/+^ mice (n = 6), *Jnk1* ^−/−^ *Trp53* ^−/+^ mice (n = 14), and *Jnk2* ^−/−^ *Trp53* ^−/+^ mice (n = 8) with mammary carcinoma were examined. JNK1-deficiency and JNK2-deficiency caused reduced mammary carcinoma-free survival compared with *Trp53* ^−/+^ mice (Log-rank test, p = 0.018 and 0.039, respectively).

We performed studies of tumor-free survival of *Trp53^−/+^* mice, *Jnk1^−/−^ Trp53^−/+^* mice, and *Jnk2^−/−^ Trp53^−/+^* mice on a BALB/c strain background. Tumor development in the *Trp53^−/+^* mice was delayed compared with *Trp53^−/−^* mice (Figures 4 & 5). Interestingly, JNK1-deficiency (p = 0.026) and JNK2-deficiency (p = 0.012) caused significantly shortened tumor-free survival compared with control *Trp53^−/+^* BALB/c mice (Figure 5A). Pathological analysis demonstrated that mammary carcinoma was the most common type of tumor detected. Mammary carcinomas were detected in 43% of control mice, 70% of *Jnk1^−/−^* mice, and 53% of *Jnk2^−/−^* mice (Figure 5B). Analysis of mammary carcinoma-free survival of *Trp53^−/+^* mice, *Jnk1^−/−^ Trp53^−/+^* mice, and *Jnk2^−/−^ Trp53^−/+^* mice demonstrated that JNK1-deficiency (p = 0.018) and JNK2-deficiency (p = 0.039) significantly decreased survival compared with control *Trp53^−/+^* mice (Figure 5C). No significant difference in mammary carcinoma-free survival between JNK1-deficient mice and JNK2-deficient mice was detected (Figure 5C).

The increased mammary carcinoma detected in JNK1-deficient *Trp53^−/+^* mice was associated with a decreased incidence of hemangiosarcoma (Figure 5B). No hemangiosarcomas were detected in JNK2-deficient *Trp53^−/+^* mice (Figure 5B). These changes in tumor spectrum may reflect the shortened tumor-free survival of *Trp53^−/+^* mice (Figure 5A,C).

Together, these data indicate that JNK1 and JNK2 are not required for mammary carcinoma development in the *Trp53^−/+^* BALB/c mouse model of breast cancer. However, both JNK1 and JNK2 can influence breast cancer development. It appears that JNK can contribute to tumor suppression.

## Discussion

### JNK1 and JNK2 are not required for the development of mammary carcinoma in the *Trp53* BALB/c mouse model

JNK plays a critical role in the development of some forms of cancer [1]. Thus, carcinogen-induced hepatocellular carcinoma [5] and BcrAbl-induced lymphoma [4] are strongly suppressed in *Jnk1^−/−^* mice and carcinogen-induced skin cancer is suppressed in *Jnk2^−/−^* mice [6]. Moreover, studies of glioblastoma, prostate cancer, and lung carcinoma cell lines have identified important roles for JNK2 [7]–[10]. Together, these data confirm that JNK plays an important role in cancer development.

The results of this study suggest that JNK may play a different role in mammary carcinogenesis because neither JNK1-deficiency nor JNK2-deficiency in the *Trp53* BALB/c mouse model caused a reduction in the incidence of mammary carcinoma. This observation strongly contrasts with the finding that JNK-deficiency can markedly suppress hepatocellular carcinoma, lymphoma, and skin cancer [4]–[6].

Although JNK1-deficiency and JNK2-deficiency did not suppress mammary carcinogenesis in the *Trp53* BALB/c mouse model, we cannot exclude the possibility that deficiency of both JNK1 plus JNK2 might reduce the formation of mammary carcinoma. Indeed, the *Jnk1* and *Jnk2* genes may have partially redundant functions [18], [20]–[22]. Studies of compound mutants with disruption of *Jnk1* plus *Jnk2* are required. The early embryonic lethal phenotype of *Jnk1^−/−^ Jnk2^−/−^* mice [23] makes such studies difficult. Nevertheless, the effect of compound JNK-deficiency on mammary carcinoma development needs to be tested in future studies.

### JNK and tumor suppression

The analysis of JNK1-deficiency and JNK2-deficiency in the *Trp53* BALB/c mouse model of mammary carcinoma development demonstrates that neither JNK1 nor JNK2 is required for breast tumorigenesis (Figures 5). In contrast, the mammary carcinoma-free survival of both *Jnk1^−/−^* mice and *Jnk2^−/−^* mice was significantly reduced compared with control mice (Figure 5C). These data suggest that JNK may have a tumor suppressor role in breast cancer. This conclusion is consistent with the observation that JNK2-deficiency increases breast cancer in a transgenic mouse model with expression of polyoma virus T antigen [24]. Moreover, human genetic analysis has identified mutations in the JNK signaling pathway in breast cancer that correlate with tumor suppression and metastasis [3]. Specifically, loss-of-function mutations in *MKK4*, a human gene that encodes an activator of JNK, is mutated at low frequency in human breast cancer [25], [26], [27], [28]. It is likely that JNK1-deficiency and JNK2-deficiency in the mouse may phenocopy the effects of *MKK4* gene mutation on breast cancer in humans. The molecular mechanism of tumor suppression by the JNK signaling pathway is unclear, but may be related to a requirement of JNK for genetic stability [24]. Indeed, it has been reported that genes that encode DNA repair enzymes are over-represented as targets of JNK pathway signaling [29]. A role for JNK in the maintenance of genetic stability is also consistent with the finding that a dominant genetic trait in the *Trp53^−/+^* BALB/c mouse model of mammary carcinogenesis is loss of heterozygosity at the *Trp53* locus [30]. This observation indicates that the formation of mammary carcinoma in JNK-deficient mice may be caused by accelerated loss of heterozygosity of tumor suppressor genes.

### Conclusions

We tested the hypothesis that JNK1 or JNK2 plays a critical role during breast cancer development. We found that neither JNK1 nor JNK2 is required for mammary carcinoma in the *Trp53* BALB/c mouse model. Breast tumor-free survival was significantly reduced by JNK1-deficiency or JNK2-deficiency. These data suggest that that JNK1 and JNK2 may play a role in mammary carcinoma suppression.
